# Theta‐gamma phase‐amplitude coupling in auditory cortex is modulated by language proficiency

**DOI:** 10.1002/hbm.26250

**Published:** 2023-02-27

**Authors:** Mikel Lizarazu, Manuel Carreiras, Nicola Molinaro

**Affiliations:** ^1^ BCBL, Basque center on Cognition, Brain and Language San Sebastian Spain; ^2^ Laboratoire de Sciences Cognitives et Psycholinguistique, Département d'Etudes Cognitives, Ecole Normale Supérieure, EHESS, CNRS PSL University Paris France; ^3^ Ikerbasque, Basque Foundation for Science Bilbao Spain

**Keywords:** brain oscillations, cross‐frequency coupling, foreign language learning, magnetoencephalography, top‐down modulations

## Abstract

The coordination between the theta phase (3–7 Hz) and gamma power (25–35 Hz) oscillations (namely theta‐gamma phase‐amplitude coupling, PAC) in the auditory cortex has been proposed as an essential neural mechanism involved in speech processing. However, it has not been established how this mechanism is related to the efficiency with which a listener processes speech. Speech processing in a non‐native language offers a useful opportunity to evaluate if theta‐gamma PAC is modulated by the challenges imposed by the reception of speech input in a non‐native language. The present study investigates how auditory theta‐gamma PAC (recorded with magnetoencephalography) is modulated in both native and non‐native speech reception. Participants were Spanish native (L1) speakers studying Basque (L2) at three different levels: beginner (Grade 1), intermediate (Grade 2), and advanced (Grade 3). We found that during L2 speech processing (i) theta‐gamma PAC was more highly coordinated for intelligible compared to unintelligible speech; (ii) this coupling was modulated by proficiency in Basque being lower for beginners, higher for intermediate, and highest for advanced speakers (no difference observed in Spanish); (iii) gamma power did not differ between languages and groups. These findings highlight how the coordinated theta‐gamma oscillatory activity is tightly related to speech comprehension: the stronger this coordination is, the more the comprehension system will proficiently parse the incoming speech input.

## Graphical abstract

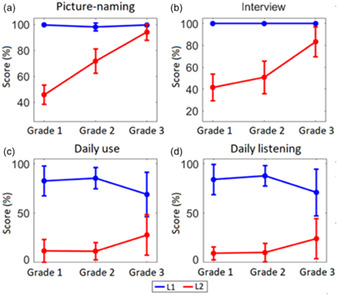

## 1 INTRODUCTION

Speech comprehension relies on the synchronization between the temporal modulations of speech and the cortical activity in the auditory cortex at multiple time scales, a multiplexing synchronization mechanism defined as cortical tracking of speech (Giraud & Poeppel, 2012; Greenberg et al., 2003; Gross et al., 2013; Molinaro & Lizarazu, 2018; Poeppel, 2003; Poeppel et al., 2008; Rosen, 1992). Temporal modulations of speech carry acoustic information that is critical for decoding auditory and linguistic information. From a theoretical perspective, speech rhythms at the syllabic (in the theta frequency band, ~3–7 Hz) and phonemic levels (in the low‐gamma power range, ~25–40 Hz) have been identified as cornerstones of speech comprehension. Neural activity in the auditory cortex has been observed to synchronize to these same frequencies, thus representing a “concerted neural coding of speech” (Obleser et al., 2012). While theta and gamma activity in the auditory cortex are weakly coupled at rest (Giraud & Poeppel, 2012), or while listening to reverse speech (Gross et al., 2013), they become more strongly related during speech listening. More specifically, the theta phase of the neural activity modulates the amplitude of gamma neural activity. This cross‐frequency phase‐amplitude coupling (PAC) has been interpreted as evidence that the syllabic tracking mechanisms (theta phase) modulate the phonemic sampling across time (Giraud & Poeppel, 2012; Gross et al., 2013; Hyafil et al., 2015). Theta‐gamma coupling would guarantee the parallel processing of the speech components at different timescales while preserving their mutual relationships and providing one mechanism for parsing the speech signal in discrete temporal windows.

According to this view, theta‐gamma PAC provides a plausible mechanism through which fine‐grain phonemic information could be grouped into syllabic units. However, alternative hypotheses are possible. Namely, theta‐gamma PAC could reflect unspecified auditory mechanisms at work during speech listening, that are not as active at rest (Giraud & Poeppel, 2012), and are not that efficient for processing unnatural auditory inputs (Gross et al., 2013). In our previous study, Lizarazu et al. (2019), we observed how this phenomenon is modulated by the speech rate: the coupling peaked at both theta and gamma increasing frequency peaks as the speech rate augmented. This indicates a relation between theta‐gamma PAC and speech processing, but we could not conclude if it is speech‐specific. The functional role of the theta‐gamma PAC in speech perception thus is still debated: is this coupling reflecting the speech comprehension accuracy (Riecke et al., 2018; Zoefel et al., 2018), or is it merely a stimulus‐driven epiphenomenon of speech processing not related with comprehension (Obleser et al., 2012)?

The present study analyzed whether theta‐gamma PAC parametrically contributes to sound‐to‐meaning mapping by testing if it is modulated by the level of experience in a given language. To this aim, three groups of second language learners attended to speech in both their native (L1, Spanish) and their second language (L2, Basque)—compared to a respective spectrally rotated speech condition (spectrally rotated Spanish and spectrally rotated Basque). Their task was to evaluate if a probe word was present in the previous passage or not. Importantly, three groups of learners were tested: beginners (Grade 1), intermediate (Grade 2), and advanced (Grade 3) L2 speakers. It is worth underscoring that: (i) we performed a within‐subject comparison with the same participants listening to speech in both L1 and L2; (ii) the speech stimuli were recorded by a balanced bilingual speaker, thus reducing possible acoustic differences (see Methods for further details); (iii) L1 and L2 present a highly overlapping phonetic repertoire (with very few exceptions), despite the two languages being typologically distant both syntactically and lexically. This is critical, since L1 speakers are already familiar with the L2 phonology; consequently, this population provides a critical test case to evaluate if theta‐gamma PAC is associated with proficient comprehension or not. In a previous study, Lizarazu et al. (2021), we reported syllabic cortical tracking of speech (in the theta band) to be sensitive to language proficiency: speech‐brain synchronization did not differ in the three groups for their L1 (Spanish) and was positively related to proficiency in L2 (Basque). In this study, we ask if this pattern holds for the theta‐gamma PAC, a mechanism assumed to reflect phonemic processing in the auditory cortex (Mesgarani et al., 2014). Such evidence would reinforce the idea that theta‐gamma PAC specifically contributes to proficient speech processing. Importantly, we seek to verify if gamma band power (*not* nested in the theta phase) present in the auditory cortex differs between groups and languages. One possible source of differential theta‐gamma PAC among groups could be due to the generally lower gamma power in one group of participants. Lower gamma power would then present a worse signal‐to‐noise ratio thus affecting our PAC analysis. To exclude this possibility, we checked that the gamma power was not different among groups as a first instance. Gamma power has been related to exogenous auditory processes (e.g., Pefkou et al., 2017), and no evidence of its potential relation with language proficiency has been reported. If we do not observe differences between groups on this parameter, we could unambiguously conclude that it is the temporal synchronization between the theta phase and the envelope of the gamma power that is different between groups.

We recorded the biomagnetic correlates (magnetoencephalography) and source‐reconstructed neural activity in the auditory cortex bilaterally. For each speech condition (Spanish, Basque and their unintelligible spectrally rotated versions), we used Mutual Information (MI) analysis to evaluate the coupling between the phase of low‐frequency (0.5–10 Hz) oscillation and the amplitude of high‐frequency (10–50 Hz) oscillation in auditory regions. Based on previous studies, we hypothesized that intelligible input would lead to stronger theta‐gamma PAC than unintelligible speech in both languages across all Grades. We do not expect group differences in the theta‐gamma PAC for L1 speech perception. On the other hand, if the theta‐gamma PAC is sensitive to experience with a given language, we would expect stronger theta‐gamma MI values for speech in L2 for the advanced group compared to the intermediate group, with the beginners showing the lowest scores.

## 2 METHODS

### 2.1 Participants

The present study is a reanalysis of previously published data in Lizarazu et al. (2021). Thirty‐eight right‐handed native Spanish‐speaking (L1, native language) healthy participants [Mean (M) age: 45.03; standard deviation (SD): 10.45] were included in this study. All participants had no history of neurological illness and did not report any peripheral hearing problems. All participants were studying Basque (L2, second language) at the same language center at different levels (Bai&By, https://www.baiby.com/en/): Grade 1 (beginners; *n* = 13), Grade 2 (intermediate; *n* = 13), and Grade 3 (advanced; *n* = 12). We post‐hoc computed the desired sample size with G*Power (Faul et al., 2009): Assuming a conventionally large *η* ^2^ of 0.14, (*α* error probability: 0.05, statistical power: 0.95) there is a 97% chance of correctly rejecting the null hypothesis (of no significant effect of the interaction Language by Grade) with a total of 30 participants (10 per group). Based on this we are confident that the sample size of the present study is adequate for addressing our experimental question.

Participants in Grade 1, 2 and 3 already achieved levels A1, B1 and C1, respectively, in the Common European Framework of Reference for Languages (CEFR), respectively. The three Grade groups did not differ significantly in age (Grade 1: M age = 42.46 years old, SD: 12.14; Grade 2: M age = 45.92 years old, SD: 8.69; Grade 3: M age = 47 years old, SD = 10.58) and sex (Grade 1: 5/13 Females; Grade 2: 8/13 Females; Grade 3: 6/12 Females) (Table S1). They attended an online language course with regular exercises to be performed online and revised during a meeting with the tutor every week. Participants were specifically recruited in order to have a similar number of participants per Grade. The Basque Center on Cognition Brain and Language (BCBL) ethical committee approved the experiment (following the principles of the Declaration of Helsinki) and all participants signed an informed consent form.

### 2.2 Behavioral screening

L1 and L2 skills were evaluated using the Basque, English, and Spanish Test (BEST) (De Bruin et al., 2017). The BEST consists of two parts. First, expressive vocabulary was assessed with a picture‐naming task. The test consisted of 65 pictures corresponding to non‐cognate words that had to be named in each language. Second, participants completed a 5‐min semi‐structured oral proficiency interview (Gollan et al., 2012). This interview comprised a set of questions ranging in difficulty and requiring the participant to use different types of grammatical constructions. The interview was conducted and assessed by a Spanish‐Basque bilingual linguist. The scoring ranged from 0 (“lowest level”) to 5 (“highest level”). Picture‐naming and interview scores were transformed into percentage scores. Additionally, participants also reported the percentage of daily use and listening exposure to each language. For the present study, the BEST scores in English were not relevant, and we only report the scores in Basque and Spanish.

### 2.3 Stimuli and procedure

Four types of speech stimuli were prepared: L1, L2, spectrally rotated L1, and spectrally‐rotated L2. The stimuli were a series of disconnected sentences. The L1 speech stimuli consisted of 40 meaningful Spanish sentences ranging in duration from 7.42 to 12.65 s (*M* = 9.9; SD = 1.13). Similarly, the L2 speech stimuli consisted of 40 meaningful Basque sentences ranging in duration from 7.24 to 14.73 s (*M* = 9.6; SD = 1.35). The Basque sentences were direct translations of the Spanish sentences to maintain a similar level of conceptual and grammatical complexity across the two sets of stimuli. The sentences were very simple, composed of high‐frequency words forming sentences with relatively low grammatical complexity. Sentences were made simple so that low‐proficient learners of Basque could get involved in performing the task. In both cases, the total duration of the speech was 6.4 min. Sentences were uttered by a Spanish‐Basque bilingual female who was instructed to read each sentence as clearly and naturally as possible. We evaluated the relevant rates of interest in the stimuli. The number of syllables as well as the number of phonemes per second was manually counted. Syllabic rates were evident at 5.5 and 5.89 Hz for L1 and L2, respectively. Phonemic rates were at 12.4 Hz in both languages (12.39 Hz in L1 and 12.40 Hz in L2).

**FIGURE 1:**
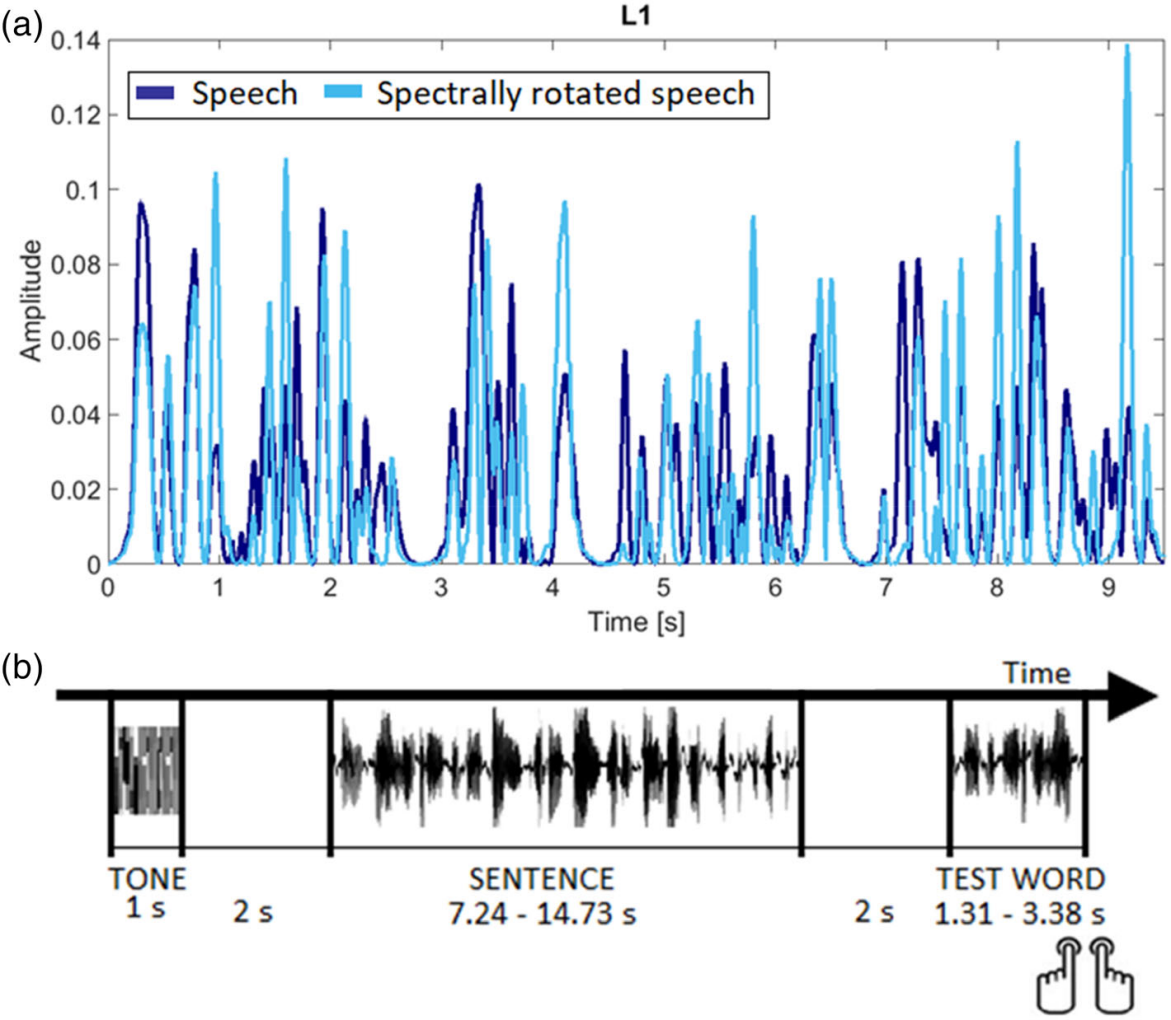
Stimuli and procedure. (a) Speech envelopes for the L1 conditions. An example of the speech envelope for a L1 sentence in the natural (dark blue) and its spectrally rotated (light blue) condition. (b) Time course of an individual trial. The structure for the L1 and L2 trials were identical.

The original Spanish and Basque speech was digitized at 44.1 kHz using a digital recorder (Marantz PMD670) and audio files (*.wav) were segmented using Praat software. The rotated speech involved a spectral inversion of the original speech. The spectrally rotated speech was produced by flipping the spectrum of the original sentences around a center frequency of 1.5 kHz by applying a custom digital implementation of the original algorithm (Blesser, 1972). Rotated speech has very similar temporal and spectral complexity to ordinary speech but is not intelligible (see Figure 1a for the L1 and Figure S1 for the L2).

During MEG recording, sentences were presented auditorily to the participants at 75–80 decibel sound pressure level. Figure 1b shows the experimental design that we followed. Each trial began with a 1 s long auditory tone (at 500 Hz tone) followed by a 2 s long silence before the sentence presentation. A probe word was presented auditorily 2 s after the end of each sentence and participants had to decide if that word was present in the previous sentence or not. During the sentence, participants were asked to fixate a white sticker attached to a front‐facing screen that was switched off. Participants answered the question by pressing the corresponding button (yes/no). After each response, the next trial was presented. Response hand was counterbalanced across participants and the presentation order of the sentences was pseudo‐randomized in four different lists. Sentence translations were never presented adjacently and, in half of the times, the Basque version of the sentence preceded the Spanish and in the other half the opposite. Participants were asked to avoid head movements and to try to blink only in between sentences. Stimuli were delivered using Presentation software (http://www.neurobs.com/). The stimuli and scripts for the MEG experiment can be found at the following link (https://osf.io/svwb8/).

### 2.4 Data acquisition

MEG data were acquired in a magnetically shielded room using the whole‐scalp MEG system (Elekta‐Neuromag, Helsinki, Finland; http://www.bcbl.eu/bcbl-facilitiesresources/meg/) installed at the BCBL. The system is equipped with 102 sensor triplets (each comprising a magnetometer and two orthogonal planar gradiometers) uniformly distributed around the head of the participant. The head position inside the helmet was continuously monitored using four Head Position Indicator coils. The location of each coil relative to the anatomical fiducials (nasion, left and right preauricular points) was defined with a 3D digitizer (Fastrak Polhemus, Colchester, VA, USA). This procedure is critical for the compensation of head movements in MEG data. Digitalization of the fiducials together with ~100 additional points evenly distributed over the scalp of the participant were used during subsequent data analysis to spatially align the MEG sensor coordinates with the T1 magnetic resonance brain images acquired on a 3T MRI scan (Siemens Medical System, Erlangen, Germany). MEG recordings were acquired continuously with a bandpass filter at 0.01–330 Hz and a sampling rate of 1 kHz. Eye movements were monitored with two pairs of electrodes in a bipolar montage placed on the external chanti of each eye [horizontal electrooculography (EOG)] and above and below the right eye (vertical EOG).

### 2.5 Data preprocessing

Signal space separation (Taulu & Kajola, 2005) was applied offline to MEG data to subtract environmental magnetic noise and correct for head movements (Maxfilter™ v2.1, Elekta Oy, Helsinki, Finland). Bad MEG channels were detected and reconstructed with an automated pipeline adapted from Bigdely‐Shamlo et al. (2015). Subsequent analyses were performed using Matlab R2010 (Mathworks, Natick, MA, USA). Heartbeat and EOG artifacts were detected using Independent Component Analysis (ICA) and linearly subtracted from recordings. The ICA decomposition was performed using the Infomax algorithm implemented in Fieldtrip toolbox (Oostenveld et al., 2011). Ocular and heartbeat ICA components were manually identified based on the spatial distribution and the temporal dynamics. Across participants, the number of heartbeat and ocular components that were removed varied between 1–4 and 1–3 components, respectively. Data were segmented using a window size of 2 s with 1 s sliding window. Furthermore, trials were visually inspected to discard any residual artifact.

### 2.6 Source activity estimation

Using the Neuromag tool MRIlab, the digitized points from the Polhemus were co‐registered to the skin surface. Individual T1‐weighted MRI images were segmented into scalp, skull, and brain components using the segmentation algorithms implemented in Freesurfer (Reuter et al., 2012). Leadfield computation was based on a three‐shell volume conductor model using a 5‐mm grid of sources defined on the MNI template brain (Gramfort et al., 2014). The template grid was transformed into individual headspace by a non‐linear space transformation algorithm (Ashburner et al., 1997; Ashburner & Friston, 1999) implemented in Statistical Parametric Mapping (SPM8, Wellcome Department of Cognitive Neurology, London, UK). The noise covariance matrix was estimated from the empty room data acquired right before bringing the participant into the MEG room. We used the noise covariance matrix to whiten the forward matrix and the data (Lin et al., 2006; Lütkenhöner, 1998). The cortical sources of the MEG signals were estimated using minimum‐norm estimates (MNE) (Hämäläinen & Ilmoniemi, 1994).

For further analysis, brain signals from predefined regions of interest were selected. The regions of interest were the left‐ and right‐hemisphere auditory cortices [Brodmann areas (BA) 41 and BA 42] (Figure S2). These regions were selected from the 3D Brodmann atlas provided with MRIcron software (available at http://www.mccauslandcenter.sc.edu/mricro/mricron).

### 2.7 Phase‐amplitude coupling analysis

There are several methods to estimate cross‐frequency coupling between two rhythms, but no single method has been selected as the gold standard. Importantly, mutual information detects both linear and nonlinear statistical dependencies between time series, whereas the more standard correlation function measures only their linear dependence. Previous studies using both simulated and empirical data have shown that mutual information is an efficient method to measure cross‐frequency coupling between two rhythms (Gross et al., 2013; Ibáñez‐Molina et al., 2020; Lizarazu et al., 2019). Ibañez‐Molina et al. (2020) showed that mutual information was sensitive to the interaction between brain oscillations in different frequency bands both in the EEG signals collected with open versus closed eyes, and in intra‐cortical brain recordings from epileptic and nonepileptic signals. Mutual Information has also been successfully used to measure brain–brain and speech–brain coupling during speech comprehension (Gross et al., 2013; Lizarazu et al., 2019). In the present study, we used mutual information to estimate the phase‐amplitude coupling (PAC) between low‐ and high‐frequency oscillations in the auditory cortex (BA41 and BA42). We followed the same procedure as in Gross et al., 2013 and Lizarazu et al., 2019. For each source in the auditory cortex, the activity was band‐pass filtered in 0.5 Hz steps between 0.5 and 50 Hz (fourth‐order Butterworth filter, forward and reverse, center frequency ± 0.5 Hz). The Hilbert transform was applied to the bandpass‐filtered signals to compute the phase and amplitude dynamics of the activity. We computed PAC between all combinations of phase (0.5–10 Hz) and amplitude (10–50 Hz) dynamics. PAC was quantified using the direct method with quadratic extrapolation for bias correction, as described in the Information Breakdown Toolbox (Magri et al., 2009). Phase and amplitude signal dynamics were quantized into 10 equipopulated bins to build marginal and joint probability distributions. Then, we calculated the average PAC value of all the sources in the left and right auditory cortex. For each language, we calculated the speech‐specific phase‐amplitude coupling (ssPAC) values as the difference between the PAC values obtained for the natural speech and the spectrally rotated speech. Ensuring ssPAC values are positive when auditory cortical activity tracked natural speech more than spectrally rotated speech, and negative otherwise.

The statistical analysis of the ssPAC values was performed in two steps. First, we identified the combination of frequency bands that showed significantly positive ssPAC values for each language across all participants. This was done with a cluster‐based permutation test that statistically compared ssPAC values to zero (Maris & Oostenveld, 2007). We repeated the permutation test on 1000 random variations of the data with a significance level of *α* = 0.05. All phase‐amplitude frequency bins were included in the test. Clusters of frequency bins showing significant ssPAC values defined the frequency bands of interest. Based on previous studies (Gross et al., 2013; Lizarazu et al., 2019), we expected to find significant positive ssPAC values between the phase of the speech envelope in the theta (~[3–7] Hz) band and the amplitude of auditory neural oscillations in the gamma (~[25–40] Hz) band (Gross et al., 2013; Lizarazu et al., 2019). Second, we investigated whether the ssPAC values changed depending on the language, level and hemisphere. The ssPAC values were averaged in the frequency bands of interest and submitted to a three‐way ANOVA on the ssPAC value, with Grade (Grade 1, 2 and 3) as between‐subject factor and Language (L1 and L2) and Hemisphere (left and right) as within‐subject factors.

### 2.8 Gamma power analysis

Changes in gamma power during speech processing were calculated for each participant and condition. For each voxel in the brain, source reconstructed time‐series were transformed into frequency power spectra by applying a Fast Fourier Transformation (FFT) to 2 s MEG data epochs. The output of this procedure was averaged across trials and frequency bins (0.5 Hz frequency resolution) in the gamma (25–40 Hz) frequency band. Then, we calculated the average gamma power value of all the sources in the left and right auditory cortex. For each language, gamma speech‐specific power (ssPow) values were calculated as the difference between the gamma power values obtained for the natural speech and the spectrally rotated speech.

We investigated whether the gamma ssPow values changed depending on the language, level, and hemisphere. For that, we run a three‐way ANOVA on the gamma ssPow values, with Grade (Grade 1, 2 and 3) as between‐subject factor and Language (L1 and L2) and Hemisphere (left and right) as within‐subject factors. As explained in the Introduction we do not expect any difference in gamma power between groups.

## 3 RESULTS

### 3.1 Behavioral screening

**FIGURE 2:**
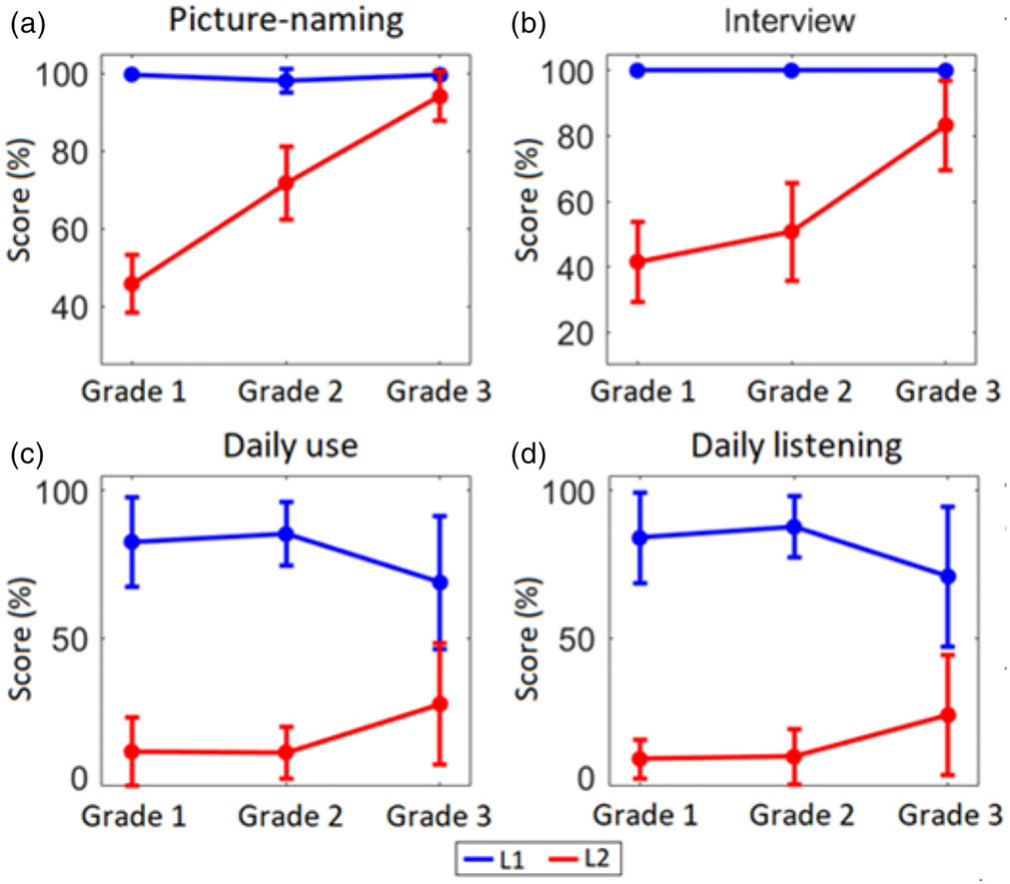
Language dominance. Mean and standard error of the scores (transformed into percentages) for L1 (Spanish) and L2 (Basque) measures across participants. (a) Picture‐naming: The test consisted of 65 pictures corresponding to non‐cognate words that had to be named in each language. (b) Interview: A personal interview with the participants to estimate their general language skill from 1 (lowest) to 5 (highest). (c) Daily use: approximate percentage of daily use of each language as reported by participants. (d) Daily listening: approximate percentage of daily listening in each language as reported by participants.

For each behavioral score (obtained before the experimental MEG session), we ran Welch's ANOVA with Language (L1 and L2) and Grade (Grades 1, 2, and 3) as factors. We observed a main effect of Language on all behavioral scores (see Figure S2 and Table S2). The Levene's test for equality of variance did not show any significant effect either in Spanish (*F*[2,34] = 0.254, *p* = .77) or in Basque (*F*[2,34] = 2.028, *p* = .15). L1 scores were higher than L2 scores across all measures. We also found a Language by Grade interaction on all scores (see Figure 2 and Table S2). Overall, the data support the contention that, as L2 learning advances, proficiency and use approach L1 levels. Post‐hoc tests of the behavioral scores are also summarized in Table S2. Regarding the word detection task performed in the MEG scanner, the percentage of correct responses for the L1 was 89.04% ± 4.15% (mean ± SD), 88.96% ± 5.4% and 88.96% ± 4.05 in Grade 1, 2 and 3, respectively. For the L2, the percentage of correct responses were 81.73% ± 7.17%, 88.96% ± 6.35% and 91.04% ± 4.05% in Grade 1, 2 and 3, respectively. We found a clear interaction between Language and Grade (*F*[2,34] = 8.76, *p* < .001). Indeed, participants answered similarly in Spanish across groups and showed increasing accuracy in Basque across Grades. Importantly, all groups answered well above chance, with accuracy higher than 80% on average. This indicates that, in the present study, participants paid attention to L2 speech stimuli and were able to recognize individual words (hence segmenting foreign speech) with more‐than‐acceptable accuracy.

### 3.2 Phase‐amplitude coupling in auditory cortex

**FIGURE 3:**
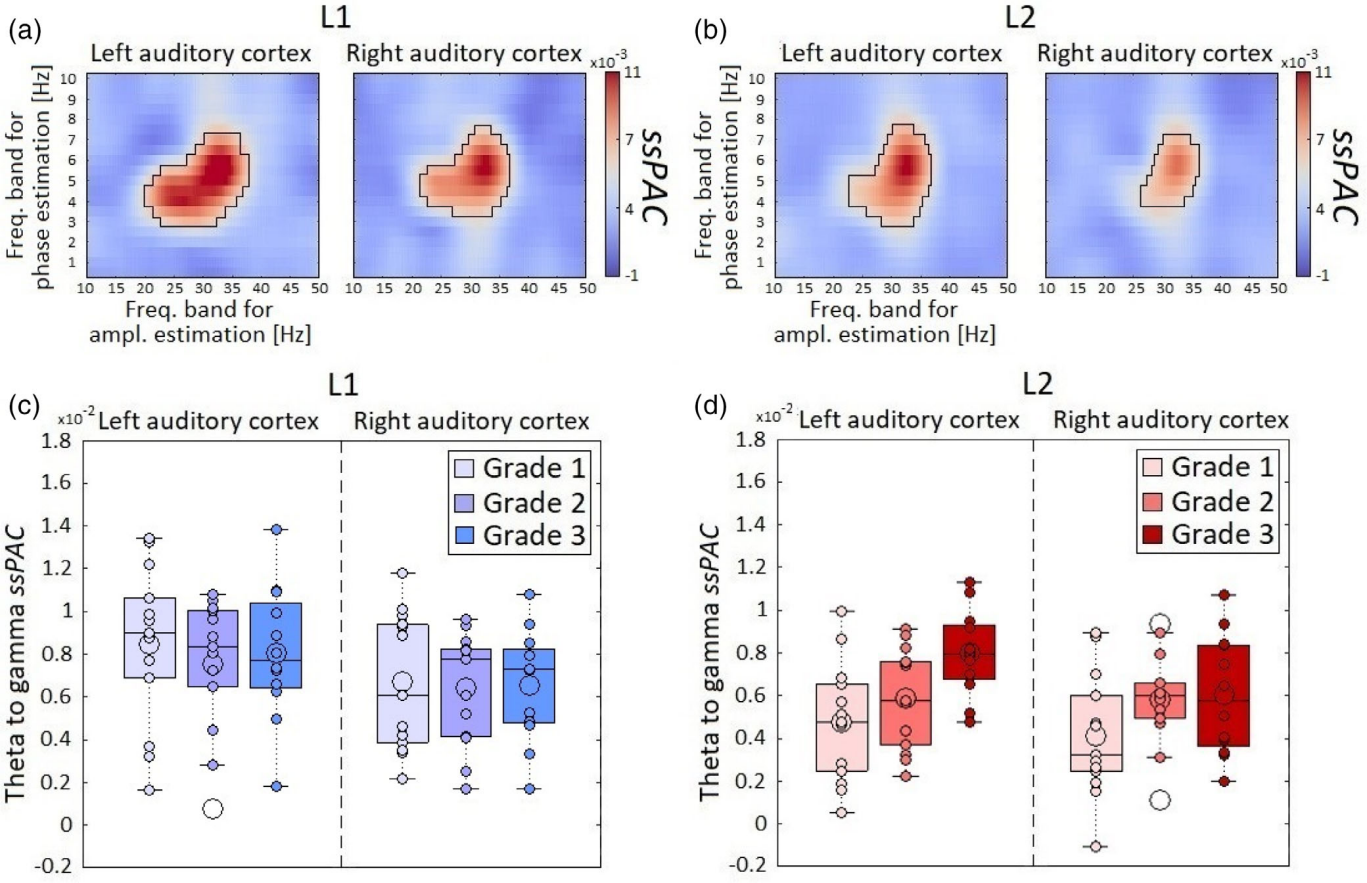
Coupling between low‐ and high‐frequency oscillations in the auditory cortex. We used mutual information to evaluate the phase‐amplitude coupling (PAC) between low‐frequency (0–10 Hz) and high‐frequency (10–50 Hz) oscillations in the auditory cortex. (a, b) Spectral distribution of the speech‐specific PAC (ssPAC) (speech—spectrally rotated speech) for each language (L1, native language; L2, second language) and auditory cortex (left and right) across all grades. Black contours highlighted significant clusters after corrected for multiple comparisons (*p* < .05, two‐tailed permutation test, cluster corrected). We also included the box plot display of the first quartile, median, mean (filled big dot) and third quartile of the theta to gamma values obtained for the (c) L1 and the (d) L2 for each language, auditory cortex and Grade (Grade 1, 2 and 3). Small filled dots represent single subject values. The extreme line represented the highest and lowest value excluding outliers. The outliers (unfilled big circles) were the points that fell more than 1.5 times the interquartile range above the third quartile or below the first quartile. Extreme lines represent the highest and lowest value (excluding outliers).

We used Mutual Information (*MI*) to evaluate the phase‐amplitude coupling (*PAC*) between low‐frequency (0–10 Hz range) oscillation and high‐frequency (10–50 Hz range) in auditory regions. PAC was calculated for each language, condition, grade, and auditory cortex (Figures S3 and S4). Then, we obtained the speech‐specific *PAC* (*ssPAC*) as the difference between the *PAC* for the natural speech and that for the spectrally rotated speech (Figure S5). We identified the combination of frequency bands showing significantly positive *ssMI* values in the left and right auditory cortex across all levels (Figure 3a,b).

For L1 (Figure 3a), *ssPAC* values were significantly positive between the phase of theta ∿(3–7 Hz) and the amplitude of gamma ∿(22–38 Hz) oscillations in left and right auditory cortex (*p* < .05, two‐tailed permutation test, cluster corrected). Similarly, for the L2 (Figure 3b), *ssPAC* values were significantly positive between the phase of theta ∿(3–7.5 Hz) and the amplitude of gamma ∿(23–37 Hz) oscillations in bilateral auditory cortices (*p* < .05, two‐tailed permutation test, cluster corrected). Negative *ssPAC* (stronger PAC for the spectrally rotated speech compared to the natural speech) were not significant.

Next, we examined with a three‐way ANOVA (Table S3) how the mean *ssPAC* values between the phase of theta (3–7.5 Hz) and the amplitude of gamma (22–38 Hz) frequency bands depend on Language (L1 and L2), learning Grade (Grade 1, 2 and 3), and Hemisphere (left and right). Figure 3c,d presents the mean *ssPAC* across participants. It clearly highlights an evolution of *ssPAC* with learning grade for L2 but not L1 that was substantiated by a main effect of Language (*F*(1,35) = 9.51, *p* < .01, $\eta_{p}^{2}$ = 0.06) and a marginal interaction of Language by Grade (*F*(2,35) = 3.11, *p* = .06, $\eta_{p}^{2}$ = 0.04); and, indeed, (i) *ssPAC* was higher for L1 compared to L2 in Grade 1 (*t*[25] = 3.79, *p* < .01, *d* = 1.08), but not in Grade 2 and in Grade 3, (ii) *ssPAC* for L2 increased with grade [Grade 3 vs. 1, *t*(23) = 3.05, *p* = .04, *d* = 0.9], and (iii) *ssPAC* for L1 did not differ between levels (|*ts*| < 0.72, *ps* = 1, |*ds*| < 0.21). The ANOVA also revealed that *ssPAC* was higher in the left than in the right auditory cortex [main effect of Hemisphere, *F*(1,35) = 7.33, *p =* .01, $\eta_{p}^{2}$ = 0.04].

**FIGURE 4:**
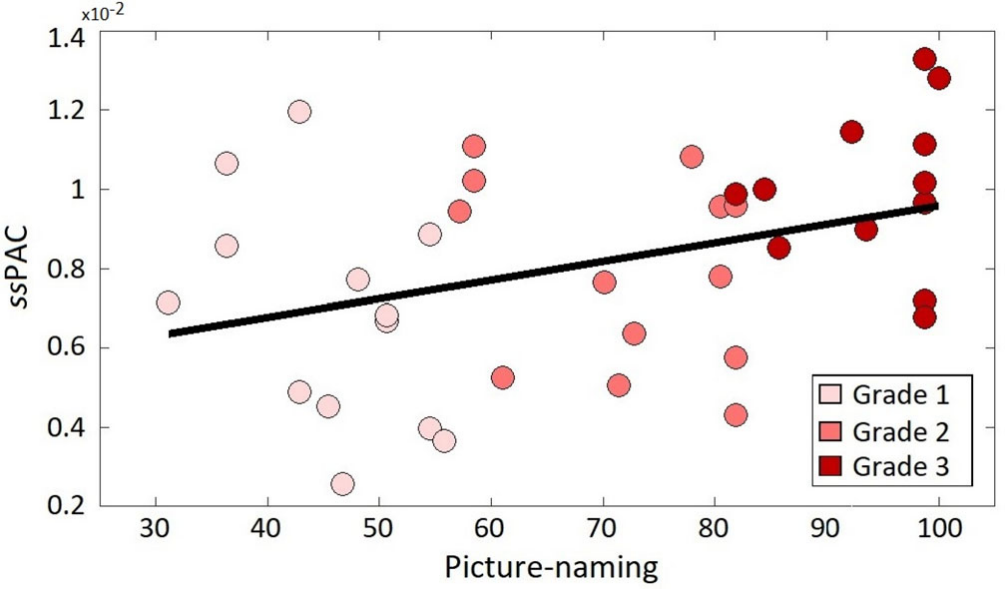
Relevance of theta to gamma coupling for L2 proficiency. Circles represent the values obtained for each participant. The black trace is the fit of the linear regression model.

Finally, we used linear regression models to evaluate the relationship between behavioral (percentage scores of picture‐naming, interview, daily use, daily listening) and theta to gamma ssPAC values in the left and right auditory cortex for L2 across grades (Table S5). Bonferroni correction was used to counteract the multiple comparisons problem. We found stronger theta to gamma ssPAC in the left auditory cortex was associated with higher scores in the picture‐naming task (*r* = 0.46, *p =* .0036) (Figure 4). No statistically significant correlations emerged for other behavioral scores. In our behavioral proficiency evaluation, picture naming was the task showing higher sensitivity to the Grade of the participants in the different groups. This is possibly determined by type of task (De Bruin et al., 2017), that employs a large number of stimuli per language (65) of increasing difficulty, providing large variability across participants. Such variability was not observed for the interview and the self‐proficiency reports (daily use, daily listening).

### 3.3 Gamma power in auditory cortex

**FIGURE 5:**
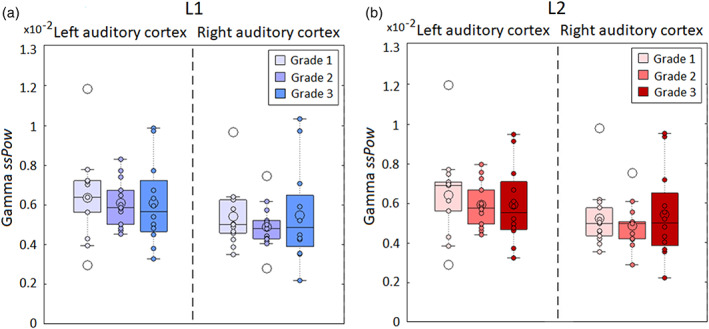
Gamma power during speech processing in the auditory cortex. Box plot display of the first quartile, median, mean (filled big dot) and third quartile of the gamma speech‐specific power (ssPow) values obtained for the (a) L1 and the (b) L2 for each language, auditory cortex and Grade (Grade 1, 2, and 3). Small filled dots represent single subject values. The extreme line represented the highest and lowest value excluding outliers. The outliers (unfilled big circles) were the points that fell more than 1.5 times the interquartile range above the third quartile or below the first quartile. Extreme lines represent the highest and lowest value (excluding outliers).

We calculated the gamma power (25–40 Hz) for each language, condition, grade and hemisphere (Figure S6). Then, we obtained the gamma speech‐specific power (ssPow) values as the difference between the gamma power for the natural speech and that for the spectrally rotated speech (Figure 5). We examined with a three‐way ANOVA (Table S4) how the gamma ssPow values in auditory regions depend on Language, learning Grade, and Hemisphere. Figure 5a,b present the gamma *ssPow values* across participants for L1 and L2, respectively. The ANOVA revealed a main effect of Hemisphere [*F*(1,35) = 27.47, *p* < .01, $\eta_{p}^{2}$ = 0.07]. Overall, *ssPow values* were higher in the left than in the right auditory cortex. No other main effect or interaction emerged.

## 4 DISCUSSION

Cross‐frequency phase‐amplitude coupling has been identified as a potential critical mechanism linking high‐frequency oscillatory activity to the slower and behaviorally relevant occurrence of external events (Canolty & Knight, 2010). The biological relevance of this phenomenon is underscored by the fact that it exists across multiple species and in different brain regions (Jensen & Colgin, 2007) and it is critical for the encoding of multi‐item sequences (Jensen & Lisman, 2005) or continuous stimuli (Schroeder et al., 2010).

During speech perception, gamma‐band oscillations reflect the working rhythm of the auditory cortex, focused on sampling the acoustic input to identify the fine‐grained properties of the stimulus such as, for example, format transitions (/*ba*/vs./*da*/) and voicing (/*ba*/vs./*pa*/; Giraud & Poeppel, 2012). Fast oscillations, such as the gamma rhythm, are thought to synchronize cell assemblies over relatively short spatial scales and this mechanism seems especially well‐suited for sensory processes (Fries et al., 2007). Importantly, this process is optimized in time depending on the speech rhythm (Lizarazu et al., 2019). This optimization seems fundamental to improving the sensory decoding of the speech input, and it is modulated in time by low‐frequency oscillatory activity (i.e., theta band oscillations) closely matching the syllable rate of the speech envelope (Giraud & Poeppel, 2012; Gross et al., 2013). In a previous study, Lizarazu et al. (2019), we observed how the pace of the speech rhythm modulates the theta‐gamma phase‐amplitude coupling: the frequency peak for both the theta phase (from ~4.7 to ~6.4 Hz) and the nested gamma power (from ~29 to ~38 Hz) depending on the increasing speech velocity. This fine‐grained modulation indicates how important the rhythm of both oscillatory components is in the theta‐gamma interaction to proficiently sample the input acoustics.

The theta‐band oscillations matching the temporal modulation of speech envelope have also been involved in the development of temporal predictions of the quasi‐rhythmic structure of speech (Arnal & Giraud, 2012). The gamma‐band sensory mechanism would be deployed based on an internal clock (or oscillator) responsible for packaging sensory stimulation into syllables. Our analysis of theta‐gamma coupling in Basque strengthens this view: in fact, the three groups that we tested did not differ in the overall gamma power observed in the auditory cortex while listening to Basque (compared to the relative spectrally rotated condition). The overall power analysis accounts for a more general oscillatory neural correlate in the gamma band, without considering its modulation by the theta phase. Only when analyzing the “synchronized” gamma oscillation, that is, the one orchestrated by the theta oscillator, the three groups differed in line with the observed proficiency in Basque and in line with their comprehension scores. Thus, the critical factor accounting for proficient speech perception is not the amount of gamma power observed in the auditory cortex, but the coordinated activity of multiple network oscillations at different time scales. This brings strong evidence on the relevance for speech comprehension of the theta‐gamma coupling *specifically*.

Hyafil et al. (2015) proposed a microcircuit computational network based on the theta‐gamma coupling mechanisms, where the timing of the temporal structure of syllables would drive the internal theta oscillator and the speech‐induced gamma activity. This model differs from previous proposals, since it does not require the assumption of a dedicated memory buffer, but a temporal integration buffer could be emulated by the theta oscillator coupled to the temporal dynamics of the incoming speech. Critically, these theta‐gamma dynamics account for the bottom‐up component of the speech perception mechanisms and, as the authors acknowledge, do not incorporate the role of higher order linguistic information in top‐down modulating sensory processing (Davis et al., 2011; Peelle et al., 2013). In the present study, we show that high‐level linguistic knowledge is critical for the efficient alignment of the oscillatory dynamics in the theta and gamma channels. In fact, the more the listener was proficient in Basque, the stronger was the theta‐gamma coupling while listening to Basque stimuli. On the contrary, these same listeners did not show this effect for Spanish, (i.e., their native language), where the coupling was high for all three groups. It is worth considering that Basque and Spanish present a highly overlapping phonemic repertoire and very similar syllabic rhythms (especially since our stimuli were produced by the same speaker, a balanced bilingual). In principle, phonemes and syllables (respectively mirrored in gamma and theta oscillatory processing) could be processed similarly (showing no modulation of PAC) in the two languages irrespective of the Basque variability in proficiency (all our participants were highly proficient in Spanish). On the opposite, we observed an almost perfect modulation of PAC based on proficiency: bilinguals were showing within‐subject PAC modulations depending on proficiency in their two languages.

An open issue concerns the identification of the neural mechanism responsible for the top‐down modulation of the theta‐gamma coupling.^1^ Previous research pointed to the role of the beta band oscillations (13–25 Hz) in top‐down control during language processing (Keitel et al., 2018; Pefkou et al., 2017; Proix et al., 2022). The role of the beta band oscillatory channel has been associated with the involvement of motor areas in temporal predictions (Keitel et al., 2018). Premotor regions were also found to modulate auditory processing when the external input poses high‐attentional demands to the perceptual system: the more challenging the comprehension of the stimulus, the stronger the recruitment of the pre‐motor cortex (Morillon et al., 2014; Morillon & Baillet, 2017; Park et al., 2018; see also Assaneo & Poeppel, 2018). A different top‐down interaction with the auditory cortex during speech processing has been described by Park et al. (2015): they observed delta band (<2 Hz) signals from the left inferior frontal cortex (and the motor regions) directed toward the auditory cortex that enhanced the coupling between the speech signal and the auditory cortex. Similar effects have been observed by our lab in the same group of bilinguals reported in the present paper (Lizarazu et al., 2021). Overall, multiple neural mechanisms have been reported as accounting for the top‐down modulation of the neural activity in the auditory cortex. Crucially, however, most of them involved temporal modulation of the auditory cortex in the delta band, a frequency band that does not match with the rhythm of the theta phase involved in the theta‐gamma coupling discussed in this paper. In Lizarazu et al. (2021, see also Park et al., 2015, for similar effects), we, however, observe theta‐band oscillations in posterior temporal regions to modulate the auditory cortex activity in the same frequency channel. Posterior temporal regions have been generally involved in lexical processing (among others, Friederici, 2011; Hagoort, 2005; Lau et al., 2008). Our idea is that the nature of this interaction lies in the fact that abstract word‐level representations influence the alignment of the auditory cortex activity to the syllabic structure of speech envelope. Hierarchically higher word‐level information would thus constrain, potentially in a predictive way, the processing of lower‐level syllable‐level processing. This, in turn, would modulate the timing of the gamma‐band activity involved in sampling the phoneme‐level speech content through theta‐gamma coupling mechanism. As reported in Lizarazu et al. (2021), the strength of the theta top‐down interaction is modulated by language proficiency, with a similar positive relation compared to the theta‐gamma coupling reported in the present paper.

The present findings thus provide critical evidence that the theta‐gamma auditory coupling represents the proficient sampling of auditory information. As reported earlier (Lizarazu et al., 2019), this mechanism flexibly adapts to the speech rate and, as we show here, it is related to the successful comprehension of the speech input. Critically, the relation between the timing of the theta phase and the timing of the gamma amplitude is the key factor enabling proficient speech decoding.

## Supporting information


**FIGURE S1.** Speech envelopes for the L2 conditions. An example of the speech envelope for a L2 sentence in the natural (red) and the spectrally rotated (yellow) condition.
**FIGURE S2.** Selection of regions of interest (ROIs). Brodmann areas 41 (red) and 42 (blue) were selected as ROIs. The brain slice in the axial plane (*Z* = 12, 14, 15, 16 in MNI coordinates) illustrates the depth of the ROIs. BA41 and BA42 in the left hemisphere of the MNI brain were also included. Figure S1: Selection of regions of interest (ROIs). Brodmann areas 41 (red) and 42 (blue) were selected as ROIs. The brain slice in the axial plane (*Z* = 12, 14, 15, 16 in MNI coordinates) illustrates the depth of the ROIs. BA41 and BA42 in the left hemisphere of the MNI brain were also included.
**FIGURE S3.** Coupling between low‐ and high‐frequency oscillations in the left and right auditory cortex for each Grade in L1 (Spanish).
**FIGURE S4.** Coupling between low‐ and high‐frequency oscillations in the left and right auditory cortex for each Grade for the L2 (Basque).
**FIGURE S5.** Spectral distribution of the speech‐specific *PAC* (*ssPAC*) (speech—spectrally rotated speech) for each language (L1, native language; L2, second language) and auditory cortex (left and right) for (A) Grade 1, (B) Grade 2 and (C) Grade 3 (D).
**FIGURE S6.** Gamma power analysis. Gamma power values for each language (L1 and L2), auditory cortex (left and right) and condition (speech and spectrally rotated speech). Box plot display of the first quartile, median, mean (filled big dot) and third quartile of the gamma speech‐specific power (ssPow) values obtained for the (A) L1 and the (B) L2 for each language, auditory cortex and Grade (Grade 1, 2, and 3). Small filled dots represent single subject values. The extreme line represented the highest and lowest value excluding outliers. The outliers (unfilled big circles) were the points that fell more than 1.5 times the interquartile range above the third quartile or below the first quartile. Extreme lines represent the highest and lowest value (excluding outliers).
**TABLE S1.** Statistical analysis of the age and sex. For each comparison between grades, we used an equivalence independent sample *t*‐test to evaluate age and sex significant differences. The equivalence region was established from −0.05 to 0.05. Abbreviations: *T*, *T*‐value; *P*, *p*‐value; *df*, degrees of freedom.
**TABLE S2.** Statistical analysis of the behavioral scores. For each test, we used a two‐way ANOVA to investigate main and interaction effects of Language (L1, native language; L2, second language) and Grade (Grades 1, 2 and 3). We performed Bonferroni post‐hoc comparisons to better characterize main effects and interactions observed in the ANOVA. Abbreviations: *N*_1_, *N*_2_ and *N*_3_ are the numbers of participants in Grades 1, 2 and 3, respectively. *F*, *F*‐value; *P*, *p*‐value; $\eta_{p}^{2}$, effect size; *T*, *t*‐value.
**TABLE S3.** Statistical analysis of the ssPAC. For each test, we used a three‐way ANOVA to investigate main and interaction effects of Language (L1, native language; L2, second language) Grade (Grades 1, 2 and 3) and Hemisphere (left, right). We performed Bonferroni post‐hoc comparisons to better characterize main effects and interactions observed in the ANOVA. Abbreviations: *N*_1_, *N*_2_ and *N*_3_ are the numbers of participants in Grades 1, 2 and 3, respectively. *F*, *F*‐value; *P*, *p*‐value; $\eta_{p}^{2}$, effect size; *T*, *t*‐value.
Table S4: Statistical analysis of the ssPow. For each test, we used a three‐way ANOVA to investigate main and interaction effects of Language (L1, native language; L2, second language) Grade (Grades 1, 2 and 3) and Hemisphere (left, right). We performed Bonferroni post‐hoc comparisons to better characterize main effects and interactions observed in the ANOVA. Abbreviations: *N*_1_, *N*_2_ and *N*_3_ are the numbers of participants in Grades 1, 2 and 3, respectively. *F*, *F*‐value; *P*, *p*‐value; $\eta_{p}^{2}$, effect size; *T*, *t*‐value.
**TABLE S5.** Relationship between behavioral and ssPAC scores. We used linear regression to evaluate the relationship between behavioral (picture‐naming, interview, daily use, daily listening) and theta to gamma ssPAC values in the left and right auditory cortex for L2 across grades. Bonferroni correction (*p*‐value threshold = 0.005) was used to counteract the multiple comparisons problem.

## Data Availability

Data sharing is not applicable to this article as no new data were created or analyzed in this study.

## Footnotes

^1^
As indicated by an anonymous Reviewer, the effect we observed could be explained without necessarily making reference to top‐down influences on PAC. It might be that, with higher proficiency, bottom‐up mapping from phonemes to syllables becomes more reliable by more precise feature encoding and more finely tuned connections between features at different levels of abstraction. Here we cannot completely exclude this hypothesis. However, since we reported evidence of feedback interactions with the auditory cortex in Lizarazu et al. (2021), we are more inclined to interpret our effects in terms of top‐down linguistic influence on low‐level auditory processing.
