# Supplementary material for: Theta‐gamma phase‐amplitude coupling in auditory cortex is modulated by language proficiency
Source: Hum Brain Mapp. 2023 Feb 27;44(7):2862–72. doi: 10.1002/hbm.26250 (PMC10089097; doi:10.1002/hbm.26250)

# Supplementary material

**Table S1: Statistical analysis of the age and sex. For each comparison between grades, we used an equivalence independent sample t-test to evaluate age and sex significant differences. The equivalence region was established from -0.05 to 0.05. Abbreviations: T, T-value; P, p-value; df, degrees of freedom.**

| **Test** | **Comparison** | **Statistic** | **T** | **df** | **P** |
| --- | --- | --- | --- | --- | --- |
| **Age** | **Grade 1 vs. Grade2:** | **T-test** | -0.84 | 24 | 0.41 |
|  |  | **Upper bound** | -0.82 | 24 | 0.79 |
|  |  | **Lowe bound** | -0.85 | 24 | 0.20 |
|  | **Grade 1 vs. Grade3:** | **T-test** | -1.01 | 23 | 0.32 |
|  |  | **Upper bound** | -1 | 23 | 0.84 |
|  |  | **Lowe bound** | -1.02 | 23 | 0.16 |
|  | **Grade 2 vs. Grade3:** | **T-test** | -0.27 | 23 | 0.79 |
|  |  | **Upper bound** | -0.26 | 23 | 0.6 |
|  |  | **Lowe bound** | -0.29 | 23 | 0.39 |
| **Sex** | **Grade 1 vs. Grade2:** | **T-test** | -1.62 | 24 | 0.26 |
|  |  | **Upper bound** | -0.91 | 24 | 0.81 |
|  |  | **Lowe bound** | -1.42 | 24 | 0.09 |
|  | **Grade 1 vs. Grade3:** | **T-test** | -0.56 | 23 | 0.58 |
|  |  | **Upper bound** | -0.32 | 23 | 0.62 |
|  |  | **Lowe bound** | -0.8 | 23 | 0.22 |
|  | **Grade 2 vs. Grade3:** | **T-test** | 0.56 | 23 | 0.58 |
|  |  | **Upper bound** | 0.8 | 23 | 0.22 |
|  |  | **Lowe bound** | 0.32 | 23 | 0.62 |

**Table S2: Statistical analysis of the behavioral scores. For each test, we used a two-way ANOVA to investigate main and interaction effects of Language (L1, native language; L2, second language) and Grade (Grades 1, 2 and 3). We performed Bonferroni post-hoc comparisons to better characterize main effects and interactions observed in the ANOVA. Abbreviations: N_1_, N_2_ and N_3_ are the numbers of participants in Grades 1, 2 and 3, respectively. F, F-value; P, p-value;** $\eta_{p}^{2}$**, effect size; T, t-value.**

| **Test** | **N_1_, N_2_, N_3_** | **ANOVA** | | | | **Post-Hoc comparison** | | | |
| --- | --- | --- | --- | --- | --- | --- | --- | --- | --- |
|  |  | **Effect** | **F** | **P** | $\eta_{p}^{2}$ | **Comparison** | **T** | **p** | $\eta_{p}^{2}$ |
| **Picture-naming** | 13, 13, 12 | LANGUAGE | 567 | <0.01 | 0.65 | L1 *vs.* L2: | 8.47 | <0.01 | 1.37 |
|  |  | GRADE | 81.66 | <0.01 | 0.82 | Grade 2 *vs.* Grade1:  Grade 3 *vs.* Grade1:  Grade 3 *vs.* Grade2: | 6.57  12.78  9.2 | <0.01  <0.01  <0.01 | 1.07  2.07  1.03 |
|  |  | LANGUAGE  by  GRADE | 135 | <0.01 | 0.31 | L1 *vs.* L2 in:  Grade 1:  Grade 2:  Grade 3:  For the L1:  Grade 2 *vs.* Grade1:  Grade 3 *vs.* Grade1:  Grade 3 *vs.* Grade2:  For the L2:  Grade 2 *vs.* Grade1:  Grade 3 *vs.* Grade1:  Grade 3 *vs.* Grade2: | 12.75  5.99  6.38  -1.91  -0.47  1.67  7.45  16.72  6.56 | <0.01  <0.01  <0.01  0.07  0.64  0.11  <0.01  <0.01  <0.01 | 2.5  1.2  1.28  -0.75  -0.19  0.67  2.92  6.7  2.63 |
| **Interview** | **13, 13, 12** | LANGUAGE | 320 | <0.01 | 0.77 | L1 *vs.* L2: | 11.44 | <0.01 | 1.86 |
|  |  | GRADE | 29.22 | <0.01 | 0.63 | Grade 2 *vs.* Grade1:  Grade 3 *vs.* Grade1:  Grade 3 *vs.* Grade2: | 1.65  7.32  5.7 | 0.32  <0.01  <0.01 | 0.27  1.19  0.93 |
|  |  | LANGUAGE  by  GRADE | 29.22 | <0.01 | 0.14 | L1 *vs.* L2 in:  Grade 1:  Grade 2:  Grade 3:  For the L1:  Grade 2 *vs.* Grade1:  Grade 3 *vs.* Grade1:  Grade 3 *vs.* Grade2:  For the L2:  Grade 2 *vs.* Grade1:  Grade 3 *vs.* Grade1:  Grade 3 *vs.* Grade2: | ---  ---  ---  ---  ---  ---  1.65  7.69  5.43 | ---  ---  ---  ---  ---  ---  0.11  <0.01  <0.01 | ---  ---  ---  ---  ---  ---  0.65  3.1  2.17 |
| **Daily use** | **13, 10, 12** | LANGUAGE | 149.5 | <0.01 | 0.08 | L1 *vs.* L2: | 11.44 | <0.01 | 1.96 |
|  |  | GRADE | 0.420 | 0.7 | 0.03 |  |  |  |  |
|  |  | LANGUAGE  by  GRADE | 3.38 | 0.05 | 0.04 | L1 *vs.* L2 in:  Grade 1:  Grade 2:  Grade 3:  For the L1:  Grade 2 *vs.* Grade1:  Grade 3 *vs.* Grade1:  Grade 3 *vs.* Grade2:  For the L2:  Grade 2 *vs.* Grade1:  Grade 3 *vs.* Grade1:  Grade 3 *vs.* Grade2: | 17.91  8.44  7.06  0.61  -1.58  -1.89  0.21  2.38  1.83 | <0.01  <0.01  <0.01  0.55  0.13  0.08  0.83  0.03  0.08 | 3.82  1.69  1.54  0.26  -0.63  -0.83  0.09  0.95  0.8 |
| **Daily Listening** | **13, 10, 12** | LANGUAGE | 117 | <0.01 | 0.76 | L1 *vs.* L2: | 10.1 | <0.01 | 1.73 |
|  |  | GRADE | 0.42 | 0.66 | 0.03 |  |  |  |  |
|  |  | LANGUAGE  by  GRADE | 3.48 | 0.04 | 0.05 | L1 *vs.* L2 in:  Grade 1:  Grade 2:  Grade 3:  For the L1:  Grade 2 *vs.* Grade1:  Grade 3 *vs.* Grade1:  Grade 3 *vs.* Grade2:  For the L2:  Grade 2 *vs.* Grade1:  Grade 3 *vs.* Grade1:  Grade 3 *vs.* Grade2: | 14.27  7.3  6.45  0.43  -1.73  -0.19  0.05  2.34  2.13 | <0.01  <0.01  <0.01  0.68  0.1  0.07  0.96  0.03  0.05 | 3.04  1.46  1.41  0.18  -0.69  -0.84  0.02  0.94  094 |

**Table S3: Statistical analysis of the ssPAC. For each test, we used a three-way ANOVA to investigate main and interaction effects of Language (L1, native language; L2, second language) Grade (Grades 1, 2 and 3) and Hemisphere (Left, Right). We performed Bonferroni post-hoc comparisons to better characterize main effects and interactions observed in the ANOVA. Abbreviations: N_1_, N_2_ and N_3_ are the numbers of participants in Grades 1, 2 and 3, respectively. F, F-value; P, p-value;** $\eta_{p}^{2}$**, effect size; T, t-value.**

| **Test** | **N_1_, N_2_, N_3_** | **ANOVA** | | | | **Post-Hoc comparison** | | | |
| --- | --- | --- | --- | --- | --- | --- | --- | --- | --- |
|  |  | **Effect** | **F** | **P** | $\eta_{p}^{2}$ | **Comparison** | **T** | **p** | $\eta_{p}^{2}$ |
| **ssPAC** | 13, 13, 12 | LANGUAGE | 9.51 | <0.01 | 0.06 | L1 *vs.* L2: | 3.08 | <0.01 | 0.52 |
|  |  | GRADE | 1.87 | 0.17 | 0.02 |  |  |  |  |
|  |  | HEMISPHERE | 7.33 | 0.01 | 0.04 | L *vs.* R: | 2.71 | 0.42 | 0.01 |
|  |  | LANGUAGE by GRADE | 3.11 | 0.06 | 0.04 | Grade 1, L1 *vs.*:  Grade 2, L1:  Grade 3, L1:  Grade 1, L2:  Grade 2, L2:  Grade 3, L2:  Grade 2, L1 *vs.*:  Grade 3, L1:  Grade 1, L2:  Grade 2, L2:  Grade 3, L2:  Grade 3, L1 *vs.*:  Grade 1, L2:  Grade 2 L2:  Grade 3 L2:  Grade 2, L2 *vs.*:  Grade 2, L2:  Grade 3 L2:  Grade 2, L2 *vs.*:  Grade 3, L2: | 0.72  0.33  3.79  2.02  0.63  -0.38  3.04  1.21  -0.07  3.36  1.65  0.3  -1.74  -3.05  -1.35 | 1  1  <0.01  0.515  1  1  0.04  1  1  0.02  0.92  1  0.86  0.04  1 | 0.207  0.1  1.08  0.58  0.19  -0.11  0.88  0.38  -0.02  0.99  0.49  0.09  -0.5  -0.9  -0.4 |
|  |  | HEMISPHERE by GRADE | 0.57 | 0.57 | <0.01 |  |  |  |  |
|  |  | LANGUAGE by HEMISPHERE | 0.54 | 0.47 | <0.01 |  |  |  |  |
|  |  | LANGUAGE by HEMISPHERE by GRADE | 0.31 | 0.74 | <0.01 |  |  |  |  |

**Table S4: Statistical analysis of the ssPow. For each test, we used a three-way ANOVA to investigate main and interaction effects of Language (L1, native language; L2, second language) Grade (Grades 1, 2 and 3) and Hemisphere (Left, Right). We performed Bonferroni post-hoc comparisons to better characterize main effects and interactions observed in the ANOVA. Abbreviations: N_1_, N_2_ and N_3_ are the numbers of participants in Grades 1, 2 and 3, respectively. F, F-value; P, p-value;** $\eta_{p}^{2}$**, effect size; T, t-value.**

| **Test** | **N_1_, N_2_, N_3_** | **ANOVA** | | | | **Post-Hoc comparison** | | | |
| --- | --- | --- | --- | --- | --- | --- | --- | --- | --- |
|  |  | **Effect** | **F** | **P** | $\boldsymbol{\eta}_{\boldsymbol{p}}^{\boldsymbol{2}}$ | **Comparison** | **T** | **p** | $\boldsymbol{\eta}_{\boldsymbol{p}}^{\boldsymbol{2}}$ |
| **ssPow** | 13, 13, 12 | LANGUAGE | 3.32 | 0.08 | <0.01 |  |  |  |  |
|  |  | GRADE | 0.21 | 0.81 | 0.01 |  |  |  |  |
|  |  | HEMISPHERE | 27.47 | <0.01 | 0.07 | L *vs.* R: | 5.24 | <0.01 | 0.56 |
|  |  | LANGUAGE by GRADE | 0.34 | 0.72 | <0.01 |  |  |  |  |
|  |  | HEMISPHERE by GRADE | 0.88 | 0.42 | <0.01 |  |  |  |  |
|  |  | LANGUAGE by HEMISPHERE | 0.7 | 0.41 | <0.01 |  |  |  |  |
|  |  | LANGUAGE by HEMISPHERE by GRADE | 1.21 | 0.3 | <0.01 |  |  |  |  |

**Table S5: Relationship between behavioral and ssPAC scores. We used linear regression to evaluate the relationship between behavioral (picture-naming, interview, daily use, daily listening) and theta to gamma ssPAC values in the left and right auditory cortex for L2 across grades. Bonferroni correction (p-value threshold=0.005) was used to counteract the multiple comparisons problem.**

| **r; p-value** | **Picture-naming** | **Interview** | **Daily use** | **Daily listening** | **MEG Word detection task** |
| --- | --- | --- | --- | --- | --- |
| **ssPAC in LAC** | 0.46; 0.0036 | 0.35; 0.03 | 0.2; 0.2666 | 0.23; 0.1985 | 0.32; 0.06 |
| **ssPAC in RAC** | 0.2; 0.2231 | 0.22; 0.1856 | 0.16;0.3687 | 0.07; 0.6797 | 0.32; 0.07 |

**Figure S1: Speech envelopes for the L2 conditions. An example of the speech envelope for a L2 sentence in the natural (red) and the spectrally rotated (yellow) condition.**

**
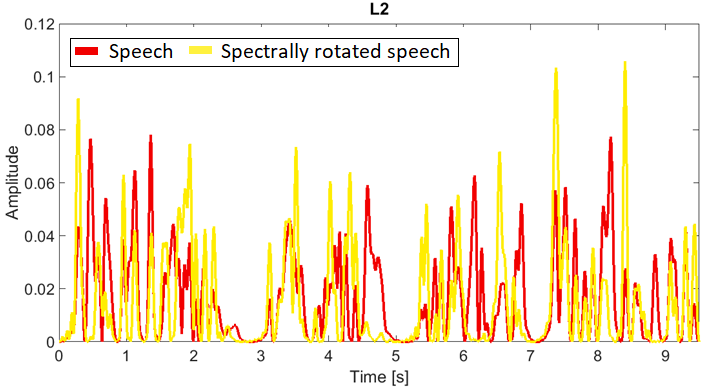
**

**Figure S2: Selection of regions of interest (ROIs). Brodmann areas 41 (red) and 42 (blue) were selected as ROIs. The brain slice in the axial plane (Z = 12, 14, 15, 16 in MNI coordinates) illustrates the depth of the ROIs. BA41 and BA42 in the left hemisphere of the MNI brain were also included. Figure S1: Selection of regions of interest (ROIs). Brodmann areas 41 (red) and 42 (blue) were selected as ROIs. The brain slice in the axial plane (Z = 12, 14, 15, 16 in MNI coordinates) illustrates the depth of the ROIs. BA41 and BA42 in the left hemisphere of the MNI brain were also included.**

**
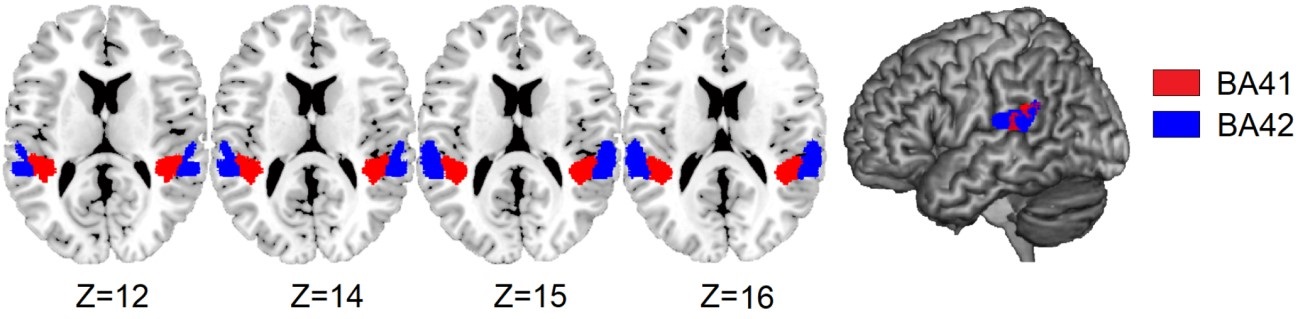
**

**Figure S3: Coupling between low- and high-frequency oscillations in the left and right auditory cortex for each Grade in L1 (Spanish).**

**
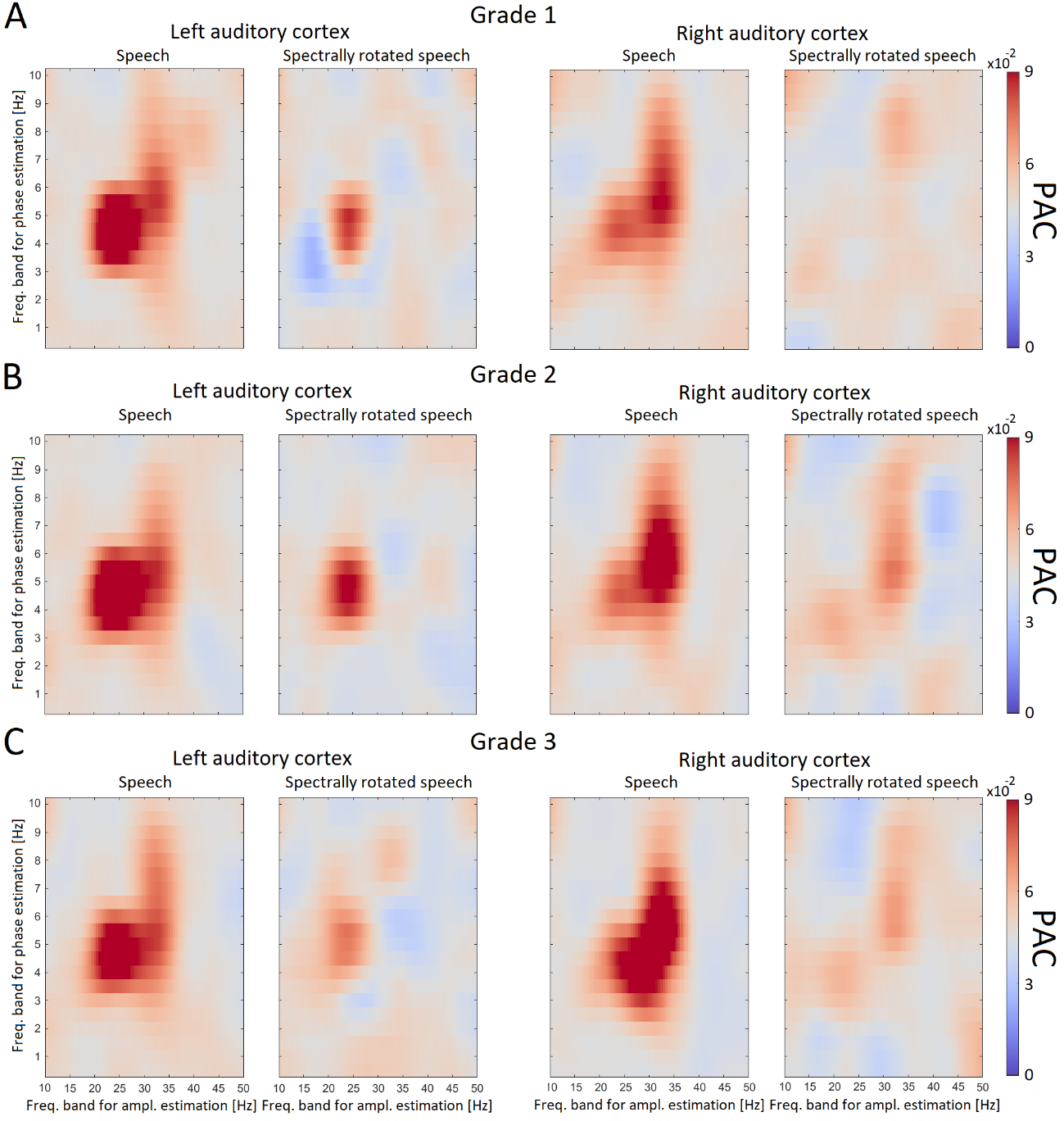
**

**Figure S4: Coupling between low- and high-frequency oscillations in the left and right auditory cortex for each Grade for the L2 (Basque).**

**
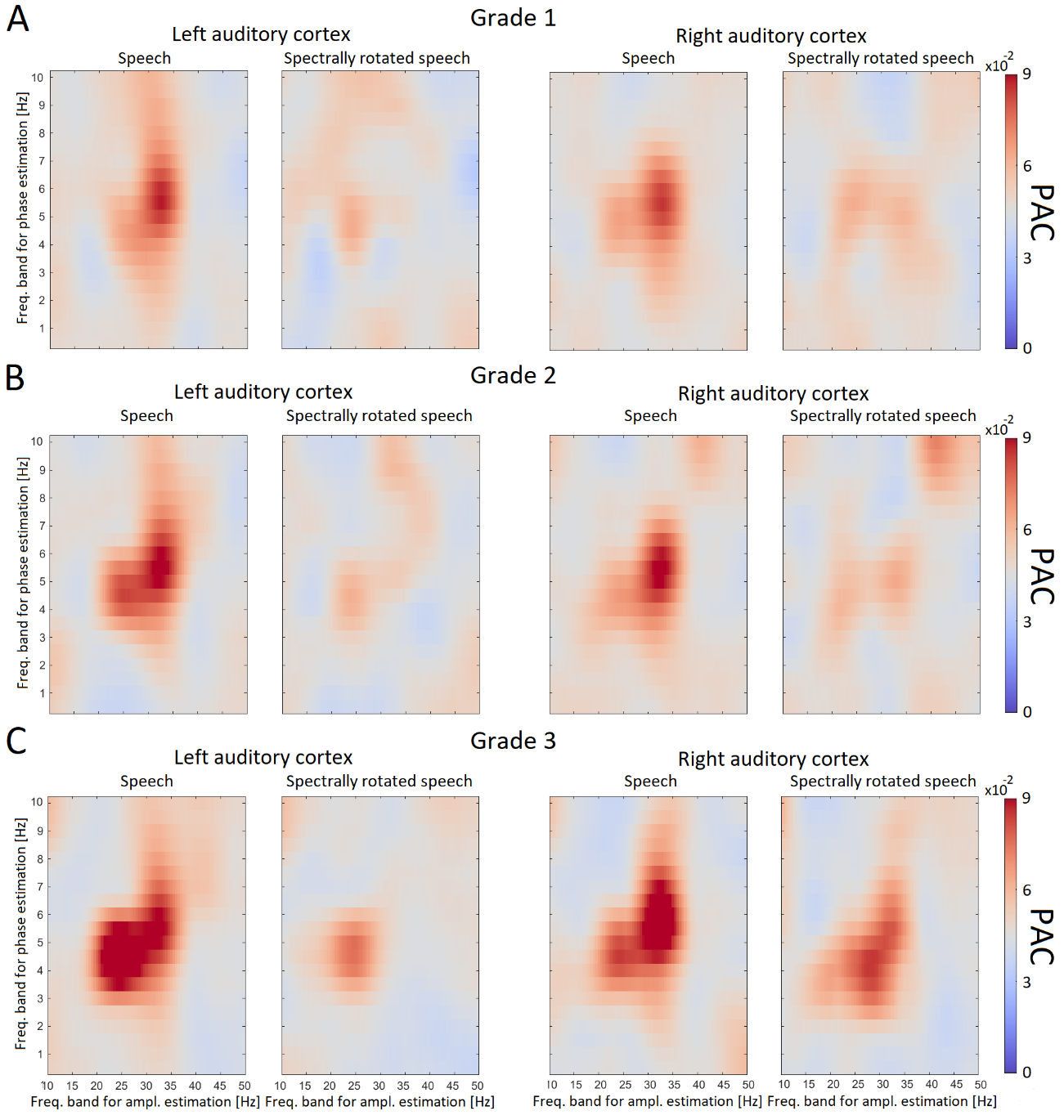
**

**Figure S5: Spectral distribution of the speech-specific *PAC* (*ssPAC*) (speech – spectrally rotated speech) for each language (L1, native language; L2, second language) and auditory cortex (left and right) for (A) Grade 1, (B) Grade 2 and (C) Grade 3 (D).**


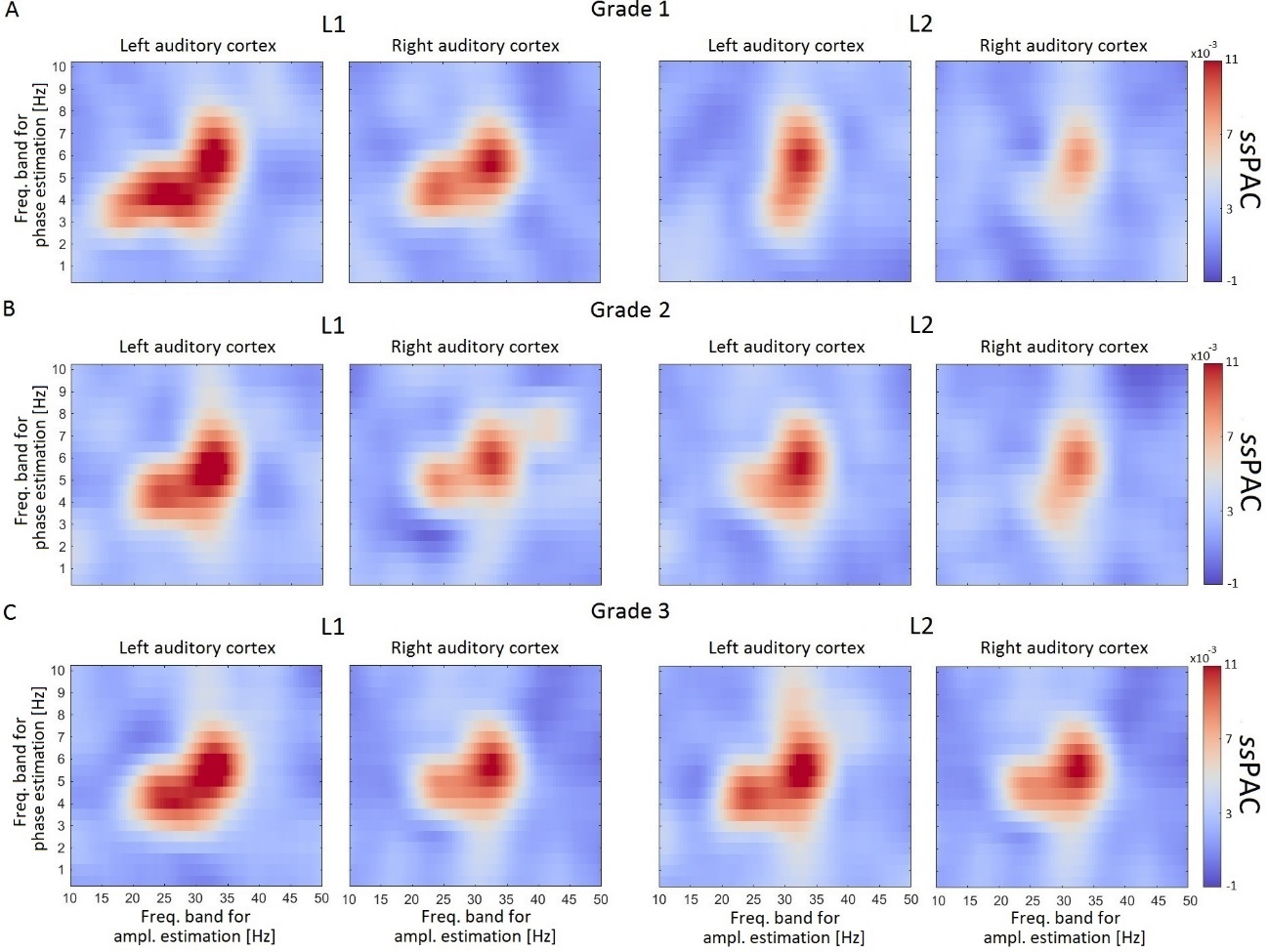


**Figure S6: Gamma power analysis. Gamma power values for each language (L1 and L2), auditory cortex (left and right) and condition (speech and spectrally rotated speech). Box plot display of the first quartile, median, mean (filled big dot) and third quartile of the gamma speech-specific power (ssPow) values obtained for the (A) L1 and the (B) L2 for each language, auditory cortex and Grade (Grade 1, 2, and 3). Small filled dots represent single subject values. The extreme line represented the highest and lowest value excluding outliers. The outliers (unfilled big circles) were the points that fell more than 1.5 times the interquartile range above the third quartile or below the first quartile. Extreme lines represent the highest and lowest value (excluding outliers).**


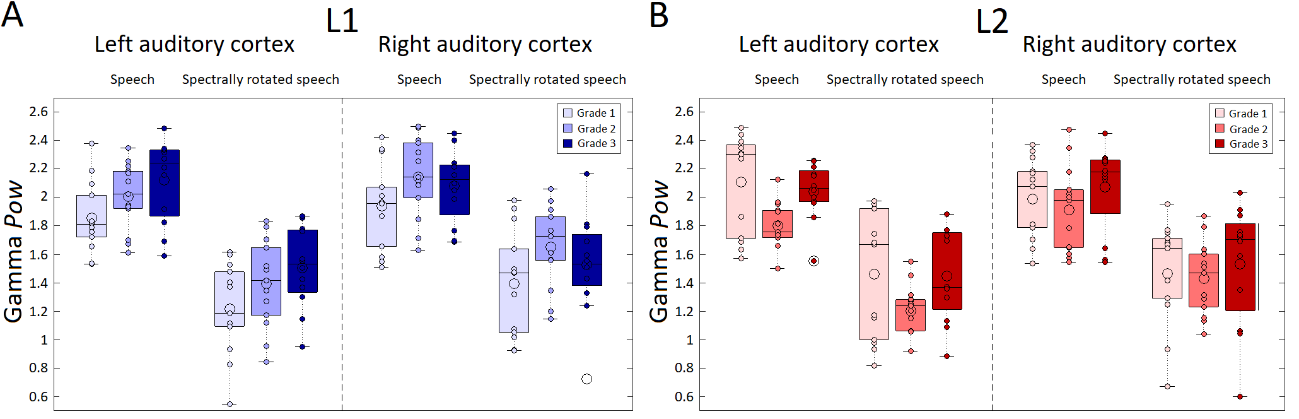

Supplement: Supplementary file 1 — FIGURE S1. Speech envelopes for the L2 conditions. An example of the speech envelope for a L2 sentence in the natural (red) and the spectrally rotated (yellow) condition. FIGURE S2. Selection of regions of interest (ROIs). Brodmann areas 41 (red) and 42 (blue) were selected as ROIs. The brain slice in the axial plane (Z = 12, 14, 15, 16 in MNI coordinates) illustrates the depth of the ROIs. BA41 and BA42 in the left hemisphere of the MNI brain were also included. Figure S1: Selection of regions of interest (ROIs). Brodmann areas 41 (red) and 42 (blue) were selected as ROIs. The brain slice in the axial plane (Z = 12, 14, 15, 16 in MNI coordinates) illustrates the depth of the ROIs. BA41 and BA42 in the left hemisphere of the MNI brain were also included. FIGURE S3. Coupling between low‐ and high‐frequency oscillations in the left and right auditory cortex for each Grade in L1 (Spanish). FIGURE S4. Coupling between low‐ and high‐frequency oscillations in the left and right auditory cortex for each Grade for the L2 (Basque). FIGURE S5. Spectral distribution of the speech‐specific PAC (ssPAC) (speech—spectrally rotated speech) for each language (L1, native language; L2, second language) and auditory cortex (left and right) for (A) Grade 1, (B) Grade 2 and (C) Grade 3 (D). FIGURE S6. Gamma power analysis. Gamma power values for each language (L1 and L2), auditory cortex (left and right) and condition (speech and spectrally rotated speech). Box plot display of the first quartile, median, mean (filled big dot) and third quartile of the gamma speech‐specific power (ssPow) values obtained for the (A) L1 and the (B) L2 for each language, auditory cortex and Grade (Grade 1, 2, and 3). Small filled dots represent single subject values. The extreme line represented the highest and lowest value excluding outliers. The outliers (unfilled big circles) were the points that fell more than 1.5 times the interquartile range above the third quartile or below the first quartile. Extreme [file HBM-44-2862-s001.docx]
